# Agreement of Single-Frequency Electrical Bioimpedance in the Evaluation of Fat Free Mass and Fat Mass in Peritoneal Dialysis Patients

**DOI:** 10.3389/fnut.2021.686513

**Published:** 2021-05-31

**Authors:** Nayrana Soares do Carmo Reis, Francieli Cristina Delatim Vaninni, Maryanne Zilli Canedo Silva, Rogério Carvalho de Oliveira, Fabrício Moreira Reis, Fabiana Lourenço Costa, Luis Cuadrado Martin, Pasqual Barretti

**Affiliations:** Internal Medicine Department, Botucatu Medical School, São Paulo State University, Botucatu, Brazil

**Keywords:** nutrition, dialysis, peritoneal dialysis, electrical bioimpedance, dual energy x-ray absorbsiometry

## Abstract

**Background:** Protein-energy wasting is related to impairment of quality of life and lower survival of end-stage kidney disease (ESKD) patients. The evaluation of body composition, especially fat free mass (FFM) and fat mass (FM), is important for the prediction of outcomes in these individuals. The aim of this study was to compare the FFM and FM measurements obtained by single-frequency bioimpedance (SF-BIA) and by a multiple frequency bioimpedance (MF-BIA) device, using dual energy X-ray absorptiometry (DXA) peritoneal dialysis (PD) patients.

**Methods:** This was a cross-sectional study involving adult patients undergoing regular PD, in which we performed SF-BIA, MF-BIA, and DXA at the same visit. To compare the bioimpedance values with DXA, we used: Person correlation (*r*), intraclass correlation coefficient (ICC), and Bland-Altman concordance analysis.

**Results:** The sample consisted of 50 patients in the PD, with mean age of 55.1 ± 16.3 years. Both bioimpedance methods showed a strong correlation (*r* > 0.7) and excellent reproducibility (ICC > 0.75) compared to DXA. According to the Bland-Altman diagram, SF-BIA showed agreement in body compartment measurements, with no proportionality bias (*p* > 0.05), without systematic bias for FFM (−0.5 ± 4.9, 95% CI −1.8 to 0.9, *p* = 0.506), and for FM (0.3 ± 4.6, *p* = 0.543). MF-BIA did not present a proportionality bias for the FFM, but it underestimated this body compartment by 2.5 ± 5.4 kg (*p* = 0.002). In addition, MF-BIA presented proportionality bias for FM.

**Conclusion:** SF-BIA was a more accurate assessing method than MBIA for FFM and FM measurements in PD patients. Because it is a low-cost, non-evaluator-dependent measurement and has less systematic bias, it can also be recommended for fat mass and free-fat mass evaluation in PD patients.

## Introduction

Protein-energy wasting (PEW) is a common condition in end-stage kidney disease (ESKD) patients. PEW is reported in 8–54% of dialysis patients and is strongly associated with adverse clinical outcomes, as an increased hospitalization rate and lower survival and has prevention and deceleration of difficult management in these individuals (1, 2). On the other hand, higher body mass index (BMI) values have been associated with better outcomes, in contrast to the association in the general population. This phenomenon has been referred to as the “obesity paradox” (3). However, the causes of this possible protective effect are still unclear since BMI does not provide accurate information about body composition or which compartment has the greatest protective effect, and this measure is likely to perform worse in dialysis patients than in the general population (4–6).

This context highlights the importance of assessing body composition to monitor and predict outcomes in ESKD patients. There are several tools for assessing body composition, with emphasis on dual energy X-ray absorptiometry (DXA), which is considered a reference method capable of more reliably estimating bone, fat, and muscle mass (7, 8). However, few clinics have access to this method, since it requires radiological medicine facilities, generating a high cost for its routine use (9).

Among the most accessible methods are electrical bioimpedance (BIA) analyses. It consists of a non-invasive and relatively low-cost method, which is easy-to-use and portable, and does not require a skilled operator, allowing reproducible results. However, this measurement can be influenced by factors related to ESKD, such as hydration status (8, 10).

BIA methods can be classified into two main categories: single-frequency BIA (SF-BIA) and multiple frequency BIA (MF-BIA). SF-BIA normally operates at a frequency of 50 kHz. This frequency and the impedance are directly proportional the total amount of body water and allows, subsequently, to establish estimates of fat-free mass. In this model, the body is divided into two parts: fat mass (FM) and fat free mass (FFM), with FM defined indirectly as the difference between body weight and FFM. This model assumes that FFM has a constant hydration of 73%. MF-BIA is based on the principle that analyzes of body compartments, using specific frequencies, yield more accurate results. It was found that at low frequencies the current moves around the cells, while at high frequencies the current penetrates the cells. Therefore, proposed that ECW should be estimated at low frequencies (5 kHz), while ICW should be estimated at high frequencies (1 MHz) (11). However, there is no consensus on which of these methods is more reliable for the assessment of body compartments such as fat-free mass (FFM) and fat mass (FM) in dialysis patients, especially peritoneal dialysis (PD), and contradictory results have been reported (12–16).

A recent update to the Clinical Practice Guideline for Nutrition in Chronic Kidney Disease (KDOQI) (8) reports that there is insufficient evidence to suggest the use of bioelectrical impedance to assess body composition of PD patients and recommends conducting future research in this group to determine the validity and reliability of these measurements in PD patients. Therefore, the aim of this study was to compare the FFM and FM measurements obtained by SF-BIA and a MF-BIA device, using DXA as the reference standard, in PD patients.

## Materials and Methods

This cross-sectional study was approved by our Institutional Ethics and Research Committee (CAAE 39704314.3.0000.5411) and involved adult end-stage kidney disease (ESKD) patients undergoing PD for at least 90 days at a single Brazilian university center. The sample size calculation was performed based on a pilot study, estimating a minimum correlation coefficient of 0.6 between the tested methods and DXA, with statistical power of 0.9 and an alpha error of 0.05.

Patients' enrollment occurred between January 2017 and May 2018. All eligible patients were invited to participate of the research and those included signed their informed consent term. We did not include patients under 18 years, with cardiac pacemakers, implanted defibrillators, and those with limb amputation, because these factors would make the nutritional assessments unreliable. We performed all body composition in patients without abdominal dialysate. A skilled examiner performed SF-BIA, MF-BIA, and DXA at the same time, with a maximum interval of 2 h between the assessments.

### Dual Energy X-Ray Absorptiometry (DXA)

FFM and FM were quantified by DXA using the Hologic® Discovery a device. The integrated software calculated lean mass (kg), FM (kg), bone mineral content (kg), lean mass index (kg/m2), and fat mass index (kg/m2). FFM was considered the sum of lean mass and bone mineral content.

### Electrical Bioimpedance

For the SF-BIA assessments, we used a Biodynamics® model 450, 800 μA, 50 kHz device and evaluated FFM (kg), FM (kg), and phase angle. The equations used to assess these measurements were based on the Kushner & Scholler proposals (16). We performed MF-BIA using the Fresenius Medical Care® Body Composition Monitor, and the body compartment measurements were estimated using a specific software provided by the manufacturer, whose formulas are based on those proposed by Moissl et al. (17). BCM assumes a division of body mass into 3 compartments: lean tissue mass (LTM), adipose tissue mass (ATM), and hyperhydration index (OH). In addition, it provides fat (kg) values consisting of hydration-free fat tissue. For comparison purposes with DXA (which divides the body into 2 body compartments), we initially used: FFM = LTM (kg) + OH (L) and FFM = total body weight (kg) - fat (kg); FM = ATM (kg) and FM = fat (kg). The analyzes using FFM = LTM (kg) + OH (L) and FM = ATM (kg) presented broader agreement limits, with greater need for adjustment. Therefore, our analyzes for BCM were based on the division of body compartments using the fat (kg) measurement provided by the device.

### Statistical Analysis

The results were expressed as the mean ± standard deviation, median (interquartile range) or percentage. Continuous variables with a normal distribution were analyzed using Student's *t*-test, and those with a non-normal distribution by the non-parametric Mann-Whitney test. To compare categorical variables, we used the Chi square test.

The correlation strength between the measurements was calculated using Pearson's correlation coefficient, while the agreement between the methods was evaluated using the intraclass correlation coefficient (ICC) and the Bland-Altman analysis. The following criteria were considered for ICC: ICC <0.4, poor reproducibility; 0.4 ≤ ICC <0.75, satisfactory reproducibility; and ICC ≥ 0.75, excellent reproducibility.

We used Bland-Altman analysis to determine the systematic and proportionality bias between the values obtained by the tested methods and DXA. Systematic bias was assessed using Student's *t*-test for one sample, checking if the average of the differences between the methods would be equal to “0.” The proportionality bias was assessed using linear regression to determine if the difference between the methods was biased by the magnitude of the measure, considering the difference between DXA and the tested method values as a dependent variable and the mean of them as an independent variable. The presence of proportionality bias classified the method as not in agreement with DXA.

The total population and subgroups were assessed, in which patients were divided according to sex, median age, and median BMI. The graphical representation of the Bland-Altman diagram is shown by means of dispersion diagrams. Continuous lines correspond to the average of the differences between the tested methods, and the DXA and dashed black lines represent the limits of agreement with 95% reliability (mean difference ± 2 × standard deviation).

We used a linear regression analysis to select predictors for the development of new equations aiming to quantify body compartments, in which the dependent variables were the FFM and FM values obtained by DXA. The equations were established using data from the tested methods in combination with demographic variables (age, sex, weight, and height). All analyzes were made using the IBM SPSS STATISTICS version 23 software, and the criterion of statistical significance corresponded to a *p* < 0.05.

## Results

### Demographic, Clinical, and Nutritional Characteristics

**Figure 1:**
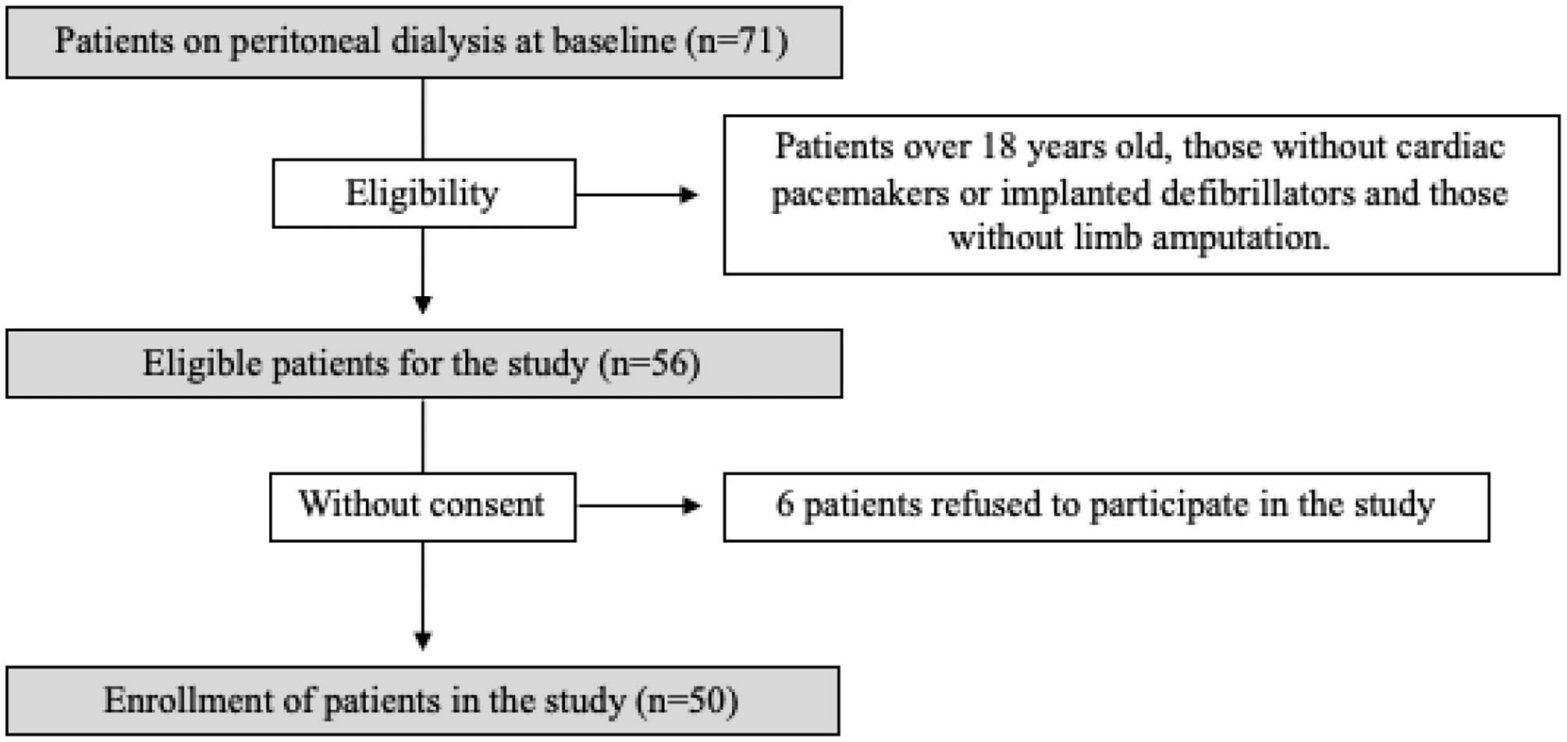
The enrollment flowchart of the study patients.

The flowchart of the patients enrolled in the study is described in Figure 1. We included 50 PD patients whose demographic and clinical characteristics are shown in Table 1. Table 2 shows the hydration status and nutritional and laboratory parameters of the study group. A strong correlation (*r* > 0.7) and excellent reproducibility (ICC ≥ 0.75) of the body composition measurements were observed between the methods tested and DXA in PD patients (Supplementary Material).

**Table 1:** Demographic and clinical characteristics of study patients.

	**Peritoneal dialysis**			
	**Total (*n* = 50)**	**Man (*n* = 23)**	**Women (*n* = 27)**	***p***
Age (y)	55.1 ± 16.3	54.4 ± 15.4	55.7 ± 17.3	0.787
**Race/ethnicity (%)**				
Caucasian	90.0	100.0	85.2	0.316
Black	2.0	0.0	3.7	
Brown	2.0	0.0	7.4	
Yellow	6.0	0.0	3.7	
Scholarity (<9 years of study)	64.0	65.2	65.4	0.990
Dialysis vintage (months)	9 (5-17)	7 (5-14)	11 (6-18)	0.163
**CKD etiology (%)**				
DM	22.0	30.4	14.8	0.658
SAH	16.0	21.7	37.5	
GCN	16.0	8.7	22.2	
Others	46.0	39.2	25.5	
SBP	140 (120–160)	140 (120–170)	140 (120–150)	0.285
DBP	80 (70–100)	80 (70–100)	80 (70–100)	0.889

*Values are presented as mean ± standard deviation, median, and interquartile range or percentage. p < 0.05. CKD, Chronic kidney disease; DM, Diabetes Mellitus; SAH, Systemic Arterial Hypertension; GCN, Glomerulus Chronic Nephritis; DBP, Diastolic Blood Pressure; SBP, Systolic Blood Pressure*.

**Table 2:** Hydration status and nutritional and laboratory parameters of study patients.

	**Peritoneal dialysis**			
	**Total (*n* = 50)**	**Man (*n* = 23)**	**Woman (*n* = 27)**	***p***
BMI (kg/m^2^)	25.8 ± 4.3	26.9 ± 4.1	24.8 ± 4.3	0.079
FFM_ SF-BIA (kg)	47.8 ± 11.6	57.0 ± 11.1	41.6 ± 6.1	<0.001
FM_ SF-BIA (kg)	20.3 ± 7.6	21.1 ± 8.3	19.6 ± 7.0	0.493
LTM_MF-BIA (kg)	37.5 ± 10.8	43.5 ± 10.0	28.8 ± 5.8	<0.001
ATM_MF-BIA (kg)	31.5 ± 11.2	31.7 ± 11.1	31.3 ± 11.4	0.897
Fat_MF-BIA (kg)	23.1 ± 8.2	23.3 ± 8.2	23.0 ± 8.4	0.901
FFM (TBM-Fat)_MF-BIA (kg)	45.8 ± 11.8	54.7 ± 10.7	38.2 ± 5.9	<0.001
FFM (LTM+OH)_MF-BIA (kg)	36.3 ± 10.8	44.3 ± 9.8	29.5 ± 5.6	<0.001
OH	0.8 ± 1.1	0.9 ± 1.1	0.7 ± 1.1	0.517
OH/ECW (%)	5.0 ± 7.2	5.0 ± 6.0	5.0 ± 8.2	0.975
FFM_DXA (kg)	48.2 ± 11.1	56.6 ± 9.2	41.1 ± 6.0	<0.001
FM_DXA (kg)	20.6 ± 6.4	21.1 ± 6.7	20.3 ± 6.3	0.647
FFM_DXA (%)	69.7 ± 6.1	72.9 ± 6.0	67.3 ± 5.0	0.001
FM_DXA (%)	30.1 ± 6.1	27.1 ± 6.0	32.7 ± 5.0	0.001
LTI_DXA (kg/m^2^)	18.0 ± 2.7	19.6 ± 2.6	16.7 ± 2.0	<0.001
FTI_DXA (kg/m^2^)	7.8 ± 2.5	7.3 ± 2.3	8.3 ± 2.5	0.184
Albumin (g/dl)	3.6 ± 0.4	3.8 ± 0.4	3.5 ± 0.4	0.012
Creatinine (mg/dl)	9.1 ± 2.9	9.9 ± 3.1	8.4 ± 2.6	0.078
Hemoglobin (g/dl)	11.1 ± 1.8	11.8 ± 2.0	10.5 ± 1.3	0.008
TC (mg/dl)	151 ± 31	142 ± 33	160 ± 27.8	0.042
CRP (mg/dl)	0.6 (0.5–0.9)	0.5 (0.5–1.1)	0.6 (0.5–0.9)	0.925

*Values are presented as mean ± standard deviation, median and interquartile range. p < 0.05. ATM, Adipose tissue mass; ECW, Extracellular water; TC, total cholesterol; DXA, dual energy x-ray densitometry; BMI, body mass index; FTI, Fat tissue index; LTI, Lean tissue index; FM, Fat mass; FFM, Fat-free mass; LTM, Lean tissue mass; OH, hyperhydration index; RCP, C-reactive protein; TG, triglyceride; TBW, total body mass; %, percentage*.

### Agreement Between SF-BIA and DXA

**Figure 2:**
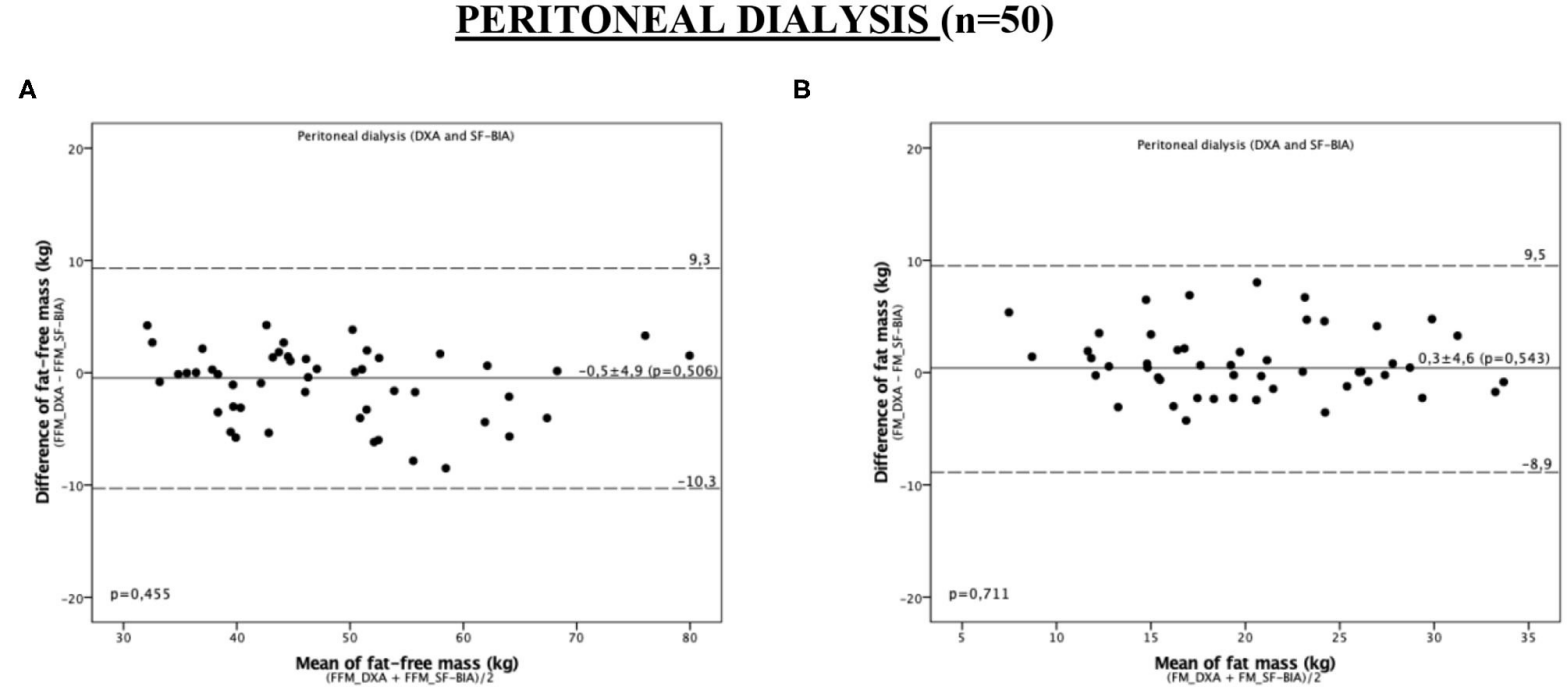
Bland-Altman plot analysis to evaluate the agreement between the methods of DXA and SF-BIA for the assessment of fat-free mass and fat mass in peritoneal dialysis patients. **(A)** FFM; **(B)** FM. The continuous lines represent the mean difference between DXA and SF-BIA and the dashed limits of agreement (mean ± 2 SD) in the 95% confidence interval.

Figure 2 shows the Bland-Altman agreement diagram for FFM and FM measured by SF-BIA and DXA. SF-BIA presented an accurate assessment of the body compartments, with no systematic or proportionality bias for FFM (−0.5 ± 4.9, *p* = 0.50) and FM (0.3 ± 4.6, *p* = 0.543) (Figure 2 and Table 3). This result was maintained in the subgroup assessment, except in the assessment of patients aged <56 years, in which the values obtained were influenced by the magnitude of the measure (proportionality bias). In addition, considering that the excess ECW is not included in the assessment of ATM, unlike DEXA, which can influence the results, we performed two Pearson's simple linear regression analysis between the difference between BCM (FFM and FM) and DEXA measurements with OH. The linear regression coefficient (r) regarding FFM and OH and FM and OH was, respectively, −0.2 (*p* = 0.115) and 0.2 (*p* = 0.093) (Supplementary Material).

**Table 3:** Agreement between the measurements obtained by DXA and single-frequency bioimpedance in the assessment of body composition in study patients.

	**Peritoneal dialysis**			
	**Systematic bias**			**Proportionalaty bias**
**DXA vs. SF-BIA**	**Bias**	**IC 95%**	***P***	
**Total (*****N*** **=** **50)**				
Fat-free mass (kg)	−0.5 ± 4.9	−1.8 a 0.9	0.506	0.455
Fat mass (kg)	0.3 ± 4.6	−0.9 a 1.7	0.543	0.053
**Man (*****n*** **=** **23)**				
Fat-free mass (kg)	−0.4 ± 6.7	−3.2 a 2.5	0.789	0.351
Fat mass (kg)	0 ± 6.2	−2.6 a 2.7	0.973	0.187
**Women (*****n*** **=** **27)**				
Fat-free mass (kg)	−0.5 ± 2.6	−1.6 a 0.5	0.298	0.822
Fat mass (kg)	0.7 ± 2.6	−0.3 a 1.7	0.178	0.127
**Age** **<** **56 years (*****n*** **=** **24)**				
Fat-free mass (kg)	−0.5 ± 6.3	−3.2 a 2.1	0.681	0.746
Fat mass (kg)	0.7 ± 6.0	−1.8 a 3.2	0.564	0.014
**Age** **≥** **56 years (*****n*** **=** **26)**				
Fat-free mass (kg)	−0.4 ± 3.2	−1.7 a 0.9	0.535	0.332
Fat mass (kg)	0.1 ± 2.8	−1.0 a 1.2	0.854	0.588
**BMI** **<** **25 kg/m**^**2**^ **(*****n*** **=** **24)**				
Fat-free mass (kg)	−0.7 ± 3.0	−2.0 a 0.6	0.263	0.168
Fat mass (kg)	0.8 ± 3.0	−0.5 a 2.1	0.204	0.055
**BMI** **≥** **25 kg/m**^**2**^ **(*****n*** **=** **26)**				
Fat-free mass (kg)	−0.2 ± 6.2	−2.7 a 2.3	0.844	0.472
Fat mass (kg)	0 ± 5.7	−2.3 a 2.3	0.980	0.128

*p < 0.05. PD, Peritoneal dialysis; DXA, dual energy x-ray densitometry; CI, Confidence interval; BMI, body mass index; SF-BIA, Single-frequency bioimpedance*.

### Agreement Between MF-BIA and DXA

**Figure 3:**
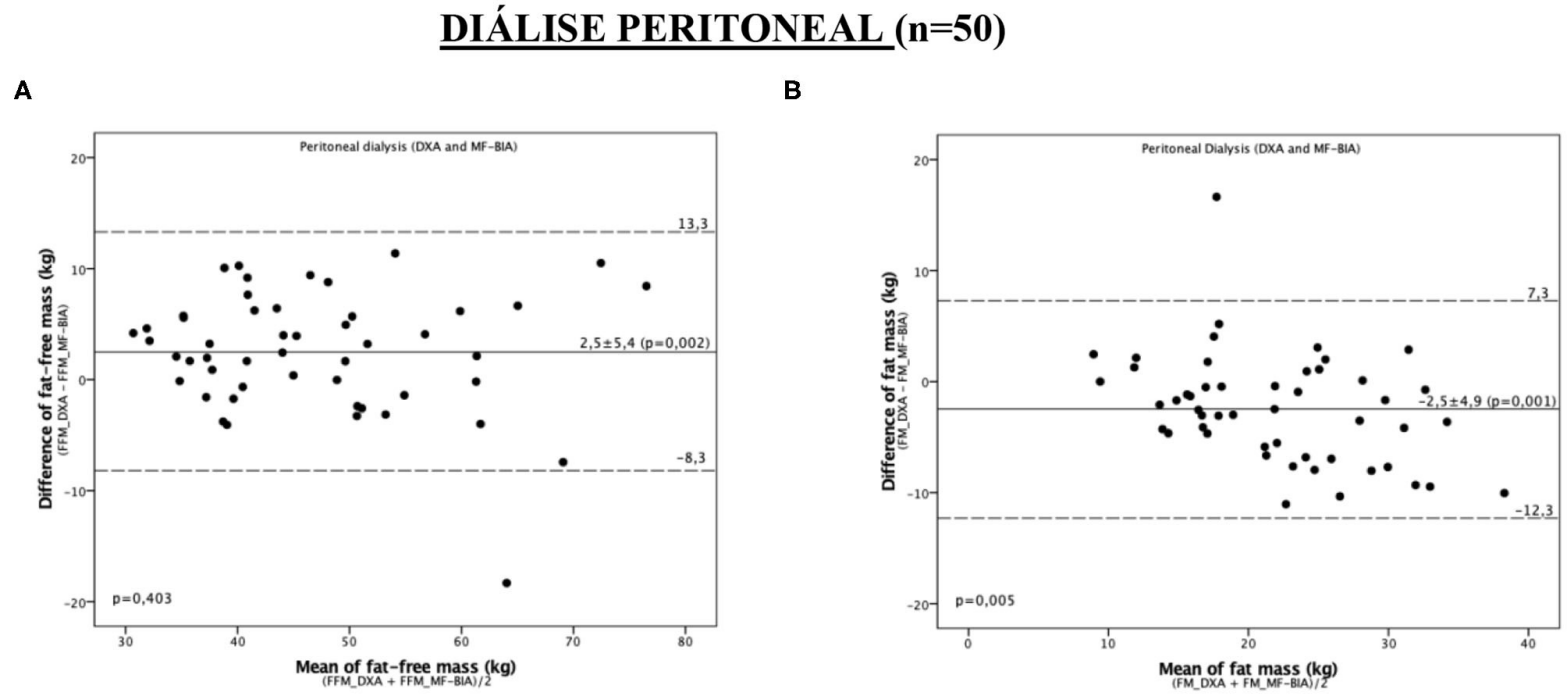
Bland-Altman plot analysis to evaluate the agreement between the methods of DXA and MF-BIA for the assessment of fat-free mass and fat mass in peritoneal dialysis patients. **(A)** FFM **(B)** FM. The continuous lines represent the mean difference between DXA and MF-BIA and the dashed limits of agreement (mean ± 2 SD) in the 95% confidence interval.

Despite not showing proportionality bias, the MF-BIA values underestimated FFM by 2.5 ± 5.4 kg in PD patients (Table 4). For this compartment, we found agreement between MF-BIA and DXA only for men and patients with BMI ≥ 25 kg/m2. For FM, we observed proportionality bias (Figure 3 and Table 4). In men, MF-BIA values agreed to the reference standard. In those aged ≥56 years, FM was overestimated by 2.9 ± 5.4 kg.

**Table 4:** Agreement between the measurements obtained by DXA and multiple-frequency bioimpedance in the assessment of body composition in study patients.

	**Peritoneal dialysis**			
	**Systematic bias**			**Proportionality bias**
**DXA vs. MF-BIA**	**bias**	**IC 95%**	***p***	
**Total (*****N*** **=** **50)**				
Fat-free mass (kg)	2.5 ± 5.4	0.9 a 4.0	0.002	0.403
Fat mass (kg)	−2.5 ± 4.9	−3.9 a −1.1	0.001	0.005
**Man (*****n*** **=** **23)**				
Fat-free mass (kg)	2.0 ± 6.6	−0.9 a 4.8	0.166	0.527
Fat mass (kg)	−2.2 ± 5.8	−4.7 a 0.3	0.087	0.205
**Women (*****n*** **=** **27)**				
Fat-free mass (kg)	2.9 ± 4.1	1.2 a 4.5	0.001	0.848
Fat mass (kg)	−2.8 ± 4.1	−4.3 a −1.1	0.002	0.003
**AGE** **<** **56 years (*****n*** **=** **24)**				
Fat-free mass (kg)	2.2 ± 5.0	0.1 a 4.3	0.041	0.748
Fat mass (kg)	–2.0 ± 4.4	−3.9 a −0.1	0.036	0.003
**AGE** **≥** **56 years (*****n*** **=** **26)**				
Fat-free mass (kg)	2.7 ± 5.8	0.4 a 5.1	0.025	0.164
Fat mass (kg)	−2.9 ± 5.4	−5.1 a −0.7	0.011	0.222
**BMI** **<** **25 kg/m**^**2**^ **(*****n*** **=** **24)**				
Fat-free mass (kg)	2.2 ± 3.1	0.9 a 3.6	0.002	0.584
Fat mass (kg)	−2.0 ± 3.1	−3.3 a −0.7	0.004	0.003
**BMI** **≥** **25 kg/m**^**2**^ **(*****n*** **=** **26)**				
Fat-free mass (kg)	2.7 ± 6.9	−0.1 a 5.5	0.058	0.307
Fat mass (kg)	−2.9 ± 6.2	−5.4 a −0.4	0.024	0.023

*p < 0.05. PD, Peritoneal dialysis; DXA, dual energy x-ray densitometry; CI, Confidence interval; BMI, body mass index; MF-BIA, Multiple frequency bioimpedance*.

### Predictors of FFM and FM

To minimize the systematic errors observed in most evaluations, we developed equations for the prediction of FFM and FM in PD patients (Chart 1), based on regression analyses (Supplementary Material). Since there was agreement in the PD patients regarding the values of FFM and FM obtained through SF-BIA and DXA, it was not necessary to construct adjustment equations for this subgroup.

**Chart 1:** Prediction equations for FFM and FM in PD patients.

**FAT-FREE MASS**
FFM_MF−BIA_ = −22,972 + 0,039 × age (years) – 3,077 × sex (0 if M; 1 if F) + 0,498 × weight (kg) + 15,886 × height (m) + 2,244 × PA (frequency 50) + 2,011 × OH.
**FAT MASS**
FM_MF−BIA_ = weight (kg) – [−22,972 + 0,039 × age (years) – 3,077 × sex (0 if M; 1 if F) + 0,498 × weight (kg) + 15,886 × height (m) + 2,244 × PA (f frequency 50) + 2,011 × OH].

*PA, Phase angle; MF-BIA, Multifrequency Bioimpedance; SF-BIA, Unifrequency Bioimpedance; M, Male; F, Female; FM, Fat mass; FFM, Fat-free mass; OH, hyperhydration index*.

## Discussion

To our knowledge, this study was the first to test the agreement of FFM and FM measurements performed by SF-BIA and MF-BIA methods against a reference standard, such as DXA, in PD patients. Our results showed that the correlations between these methods and DXA were strong, as described in previous reports (10, 18). However, the correlation coefficient alone is not sufficient to suggest agreement between methods; an adequate concordance analysis is required. Our results showed that SF-BIA can be considered reliable in FFM and FM assessment in PD patients. This finding has great importance, since nutritional status is associated with dialysis outcomes (19). Previous studies by our group have shown that BIA measurements are associated with cardiovascular outcomes (20).

Huang et al. (21) followed up patients for ~8 years, reporting that greater FFM was able to predict better patient and technique survival in PD patients. Kang et al. (22) showed an association of low appendicular mass (assessed by DXA) with all-cause mortality in incident PD patients. A recent systematic review and meta-analysis, including four studies and more than 50,000 PD patients, showed a higher mortality risk in patients with lower BMI values (23). However, the BMI *per se* does not discriminate body components such as FFM and FM, which can be estimated by more specific methods.

PD patients have less variation in hydration status due to the continuous nature of their therapy (24), which can contribute significantly to agreement of body compartments using the instruments available in clinical practice, especially when compared to HD patients, who normally retain 1–4 liters over the interdialytic interval (10).

In the evaluation of the FFM, the MF-BIA did not present proportionality bias; however, it underestimated this measure. As the error occurred systematically, the measurement of FFM by these methods can also be considered possible, provided that adjustments are made to the values obtained. Konings et al. (25) found wide limits of agreement between MF-BIA and DXA, when assessed the body composition of 40 PD patients, with a strong influence on the hydration status. Differently, there was a proportional bias between DEXA and FM, which could be a consequence of the excess ECW not being included in the assessment of FM, unlike DEXA. However, the absence of significant correlation between the differences of BCM and DEXA FM measurements and OH contradicts this possibility.

Since our results showed low agreement with MF-BIA in the body composition assessment with DXA, we constructed predictive equations to quantify FFM and FM in PD patients. The main differences in body composition measurements between BIA devices are prediction equations and alternating current frequencies. While SF-BIA analysis depends on the use of regression models, MF-BIA devices often use fit equations for a polynomial curve. In addition, each device is calibrated using its own equation and software. Therefore, despite the theoretical expectation of MF-BIA devices being more promising and reliable from a clinical perspective for the assessment of body composition, we suggest that single frequency devices are not inferior to multifrequency devices, and this has already been shown in previous studies (26). However, some adjustments in mathematical models can be applied in order to improve the agreement of the MF-BIA measures, such as the formulas we propose in our study, despite the need for validation. Predictive equations can have great relevance due to the impossibility of routine evaluation of body compartments using a reference method such as DXA due to its high complexity and high costs. However, it is important to highlight that the validation of the new equations still needs to be performed in a larger and independent population sample.

A potential limitation of our study is the absence of a gold standard in the assessment of body FFM in dialysis patients, since DXA assumes a constant hydration value. Despite this, DXA is still considered as the reference method by the KDOQI guideline (8) for assessment of body composition in ESKD patients. Its strengths are related to the moment when the evaluations were performed, with all the measurements taken on the same day by a single trained evaluator. In addition, the verification of the agreement was not limited to the evaluation of the systematic bias (as in other existing studies) but also included the objective analysis of the proportionality bias, constituting a more precise analysis.

In conclusion, the current results showed that SF-BIA agreed with DX in the evaluation of FFM and FM in PD patients, in opposite to MF-BIA measurements. Because its low-cost and being a non-examinator dependent method, SF-BIA can be recommended for the evaluation of free-fat mass and fat mass in PD patients.

## Data Availability Statement

The original contributions presented in the study are included in the article/Supplementary Material, further inquiries can be directed to the corresponding authors.

## Ethics Statement

The studies involving human participants were reviewed and approved by Institutional Ethics and Research Committee of the Botucatu Medical School, São Paulo Paulo State University (Brazil). The patients/participants provided their written informed consent to participate in this study.

## Author Contributions

NR, FV, and PB were responsible for conceptualization and methodology. NR, MS, FR, and FC performed data acquisition. NR, RO, and LM performed data analysis and interpretation. NR and FV were involved in formal analysis and wrote the original draft. NR, FV, MS, FR, LM, and PB revised and edited the manuscript. FV, LM, and PB were responsible for supervision and mentorship. All authors provided intellectual content of critical importance to the work and gave final approval of the version to be published.

## Conflict of Interest

The authors declare that the research was conducted in the absence of any commercial or financial relationships that could be construed as a potential conflict of interest.
