# Supplementary material for: Agreement of Single-Frequency Electrical Bioimpedance in the Evaluation of Fat Free Mass and Fat Mass in Peritoneal Dialysis Patients
Source: Front Nutr. 2021 May 31;8:686513. doi: 10.3389/fnut.2021.686513 (PMC8200407; doi:10.3389/fnut.2021.686513)
Supplement: Supplementary file 1 [file Table_1.DOCX]

**SUPPLEMENTARY MATERIAL**

**Pearson's Correlation Coefficients (r) between body composition measured by DXA, SF-BIA and MF-BIA in the study participants.**

|  | **PERITONEAL DIALYSIS** | | |
| --- | --- | --- | --- |
|  | **Total**  **(n=50)** | **Man**  **(n=23)** | **Woman**  **(n=27)** |
| **SINGLE-FREQUENCY BIOIMPEDANCE** | | | |
| Fat free mass (kg) | 0,91 | 0,80 | 0,90 |
| Fat mass (kg) | 0,80 | 0,67 | 0,93 |
| **MULTIPLE FREQUENCY BIOIMPEDANCE** | | | |
| Fat free mass (kg) | 0,88 | 0,79 | 0,76 |
| Fat mass (kg) | 0,80 | 0,71 | 0,89 |

Pearson's correlation test (r). p <0.001. Abbreviations: MF-BIA, Multiple frequency bioimpedance; SF-BIA, Single-frequency bioimpedance; DXA, Dual energy X-ray densitometry.

**Intraclass correlation coefficient (ICC) between the measurements obtained by DXA, SF-BIA and MF-BIA in the assessment of body composition in the study patients.**

|  | **PERITONEAL DIALYSIS** | | | |
| --- | --- | --- | --- | --- |
|  | **Man (n=35)** | **Total**  **(n=62)** | **Man (n=35)** | |
| **SINGLE-FREQUENCY BIOIMPEDANCE** | | | |  |
| Fat free mass (kg) | 0,95 | 0,88 | 0,95 | |
| Fat mass (kg) | 0,88 | 0,79 | 0,96 | |
| **MULTIPLE FREQUENCY BIOIMPEDANCE** | | | |  |
| Fat free mass (kg) | 0,93 | 0,88 | 0,81 | |
| Fat mass (kg) | 0,84 | 0,79 | 0,89 | |

Intraclass correlation coefficient (ICC). p<0,001. Abbreviations: MF-BIA, Multiple frequency bioimpedance; SF-BIA, Single-frequency bioimpedance; DXA, Dual energy X-ray densitometry

**Multiple linear regression for FFM by DXA with predictor variables in the study patients**

|  | **FAT FREE MASS - DXA** | | | | |
| --- | --- | --- | --- | --- | --- |
|  | **Nonstandard coefficients** | **Standardized coefficients** | **p** | **R^2^**  **adjusted** |  |
| **Fat free mass – MF-BIA** | | | | | |
| Constant | -22,972 |  |  |  |  |
| Age (years) | 0,039 | 0,057 |  |  |  |
| Sex | -3,077 | -0,139 |  |  |  |
| Weight (kg) | 0,498 | 0,688 | <0,001 | 0,936 |  |
| Height (m) | 15,886 | 0,132 |  |  |  |
| PA (°) | 2,244 | 0,216 |  |  |  |
| OH (L) | 2,011 | 0,200 |  |  |  |

Multiple linear regression. p<0.05. Abbreviations: PA, Phase angle; MF-BIA, Multiple frequency bioimpedance; DXA, dual energy x-ray densitometry; FFM, Fat-free mass; OH, Hyperhydration index.

**Correlation between OH and difference of the FFM and FM obtained by BIA and DXA in the study patients.**

|  | **PERITONEAL DIALYSIS** | | |
| --- | --- | --- | --- |
|  | **r** | **p** | |
| **SF-BIA – DXA** | | |  |
| Fat free mass (kg) | 0.1 | 0.546 | |
| Fat mass (kg) | 0.1 | 0.521 | |
| **MF-BIA – DXA** | | |  |
| Fat free mass (kg) | -0.2 | 0.115 | |
| Fat mass (kg) | 0.2 | 0.093 | |

p<0,001. Abbreviations: MF-BIA, Multiple frequency bioimpedance; SF-BIA, Single-frequency bioimpedance; DXA, Dual energy X-ray densitometry


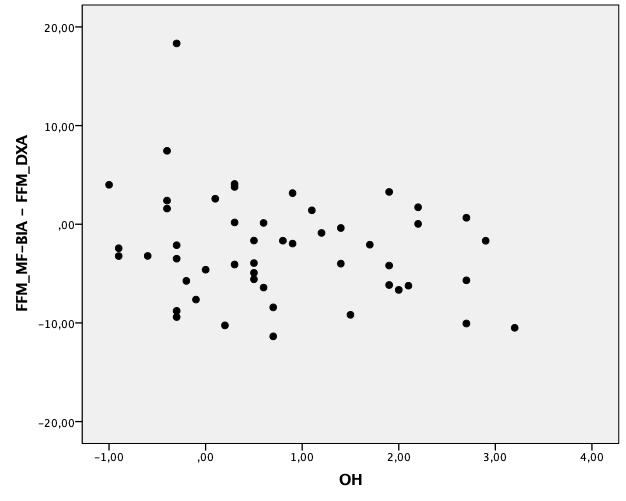


**Correlation between OH and difference of the FFM obtained by MF-BIA and DXA in peritoneal dialysis patients**


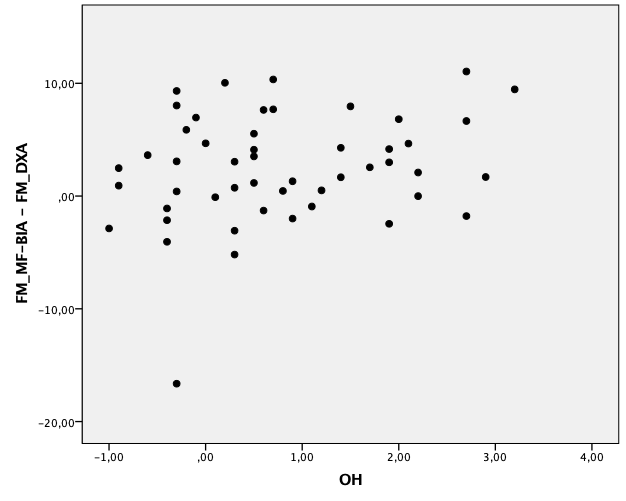


**Correlation between OH and difference of the FM obtained by MF-BIA and DXA in peritoneal dialysis patients**
